# Supplementary material for: Decoding Neural Responses to Motion-in-Depth Using EEG
Source: Front Neurosci. 2020 Dec 10;14:581706. doi: 10.3389/fnins.2020.581706 (PMC7758252; doi:10.3389/fnins.2020.581706)
Supplement: Supplementary file 1 [file Data_Sheet_1.pdf]

Supplementary Materials

Behavioural responses

A paired samples t-test was used to examine differences in the proportion of correct responses when comparing between CD and IOVD stimuli (pooled across direction). As illustrated in **Figure S1A**, participants were significantly better at discriminating the direction of CD stimuli when compared to IOVD stimuli, $t(19) = 2.269$, $p < .05$, with participants 15.9% more accurate in identifying the direction of CD stimuli. Next, we split our data into the four stimulus conditions and a four-way repeated measures ANOVA was performed to assess whether there were differences in the proportion of correct responses between the four conditions. Mauchly's test of sphericity was met ($\chi2(5) = 9.107$, $p = .107$). The ANOVA found that there was a main effect of stimulus condition on the proportion of correct responses $F(3,27) = 3.341$, $p < .05$, $\eta_p^2 = .271$, power = .693. As presented in **Figure S1B,** pairwise comparisons revealed that participants were more accurate at identifying CD towards responses when compared to IOVD towards responses ($p < .05$). No other comparisons were significant, suggesting that the increase in accuracy found in the pooled CD data when tested against the pooled IOVD data was primary driven by increased accuracy in the CD towards responses when compared to the IOVD conditions.

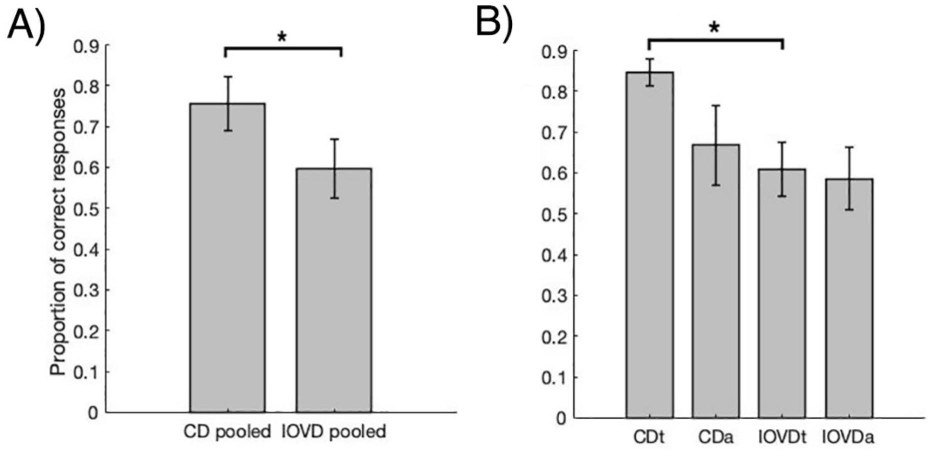

Figure S1. Bar plots of performance accuracy for identifying towards and away motion direction for MID stimuli. In panel A) we show the mean proportion of correct responses after data are pooled into CD and IOVD conditions and in B) we present the mean proportion of correct responses comparing across all four stimulus conditions. Error bars are ±1 SEM.

Decoding keyboard responses: motor responses do not contribute to decoding performance

On each trial, participants were required to respond, via the keypad, whether they perceived the stimulus as moving towards or away in depth. The signals generated from pressing one of the two keys could, potentially, influence decoding accuracy. To address this, we ran the decoder on simulated data that differed only according to how participants responded on the keyboard during the trial. Here, we pooled our data into epochs labelled with either a 'towards' or an 'away' keyboard press response. This resulted in eight pools of data, for each participant: four in which the keyboard direction response matched the stimulus, four in which it was erroneous. We then performed a decoding analysis on keyboard button press (i.e. can we decode between when participant select towards or away on the keyboard?) using datasets that contained equal numbers of 'towards' and 'away' stimuli, drawn from these eight pools. Nine participants were included in this analysis. One participant was not included as they had only a single epoch in one of these pools (i.e. they were near perfect in identifying stimulus direction). For each of the eight data pools, we bootstrapped 210 epochs by taking four random epochs from each data pool and averaging them together. Thus, each of the eight data pools was now equal in size (210 simulated epochs), with four pools containing data made from 'towards keyboard press' epochs and the other four pools containing data made from 'away keyboard press' epochs. The subsequent analysis was identical to our main decoding analysis, repeatedly bootstrapping 21 'towards keypress' epochs and 21 'away keypress' epochs for each of 1000 iterations of the classifier.

The results (**Figure S2**) show that it is not possible to decode keyboard responses independently of the stimulus, with accuracy hovering around the 50% (chance) baseline across the 125 time points. These results suggest that stimulus decoding performance is likely to be driven by MID cue properties, rather than differences in signal from the motor cortex in response to keyboard button press.

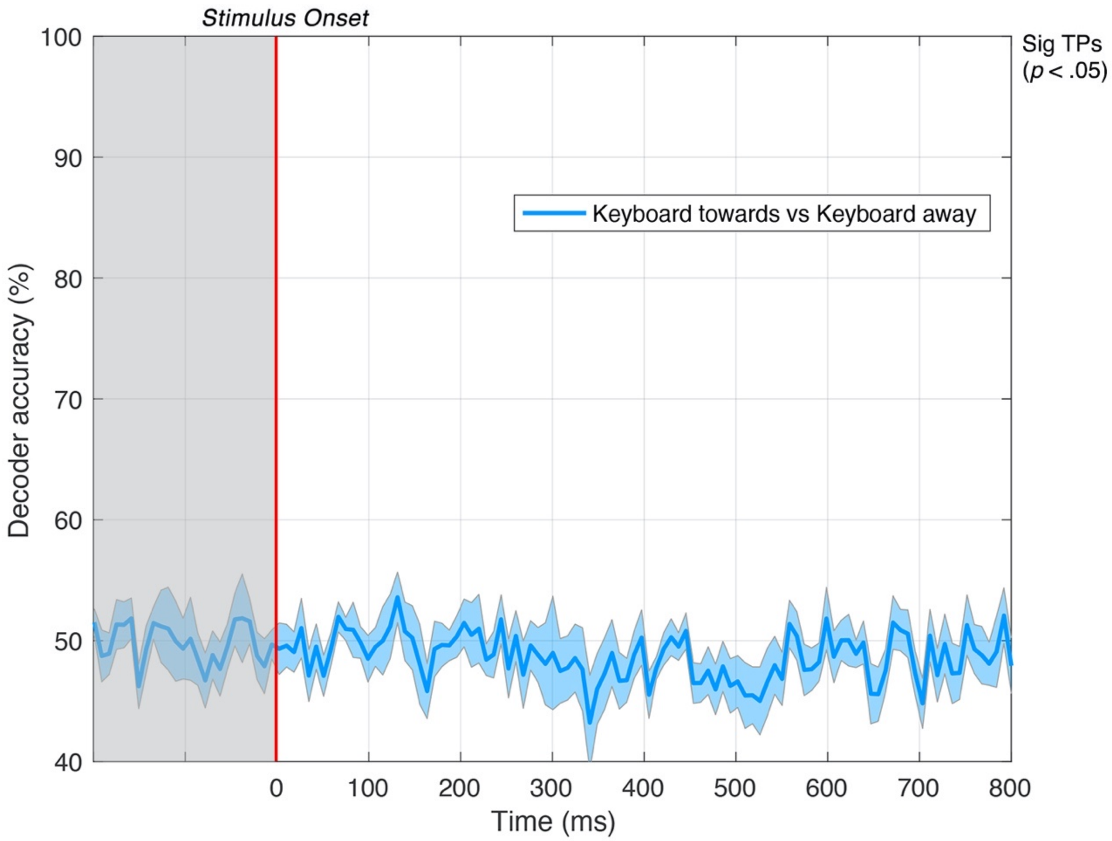

Figure S2 Pairwise decoding accuracy across 125 time points for keyboard response. Keyboard response (either pressing 2 on the keypad to indicate towards or 8 on the keypad to indicate away) cannot be accurately decoded: accuracy is at chance across all time points. Shaded error bars represent ±1SE of the bootstrapped mean (1000 iterations).

Thus, any post-stimulus decoding seen in this study cannot be accounted for by different keyboard motor responses as the decoder was unable to differentiate between towards and away keyboard responses. This has further implications. By decoding keyboard responses, we are effectively decoding the *percept* of 3D motion direction, that is, observers perceiving the stimulus either moving towards or away in depth (as well as occasional ‘button errors’). Thus, these results suggest that it is not possible to decode signals relating to the conscious percept of 3D motion direction for MID cues – although some brain regions (including motor cortex) do presumably carry such signals.

Decoding the directional perception of MID based on behavioural responses

In an additional analysis we decoded the *conscious perception* of MID direction, that is, whether the classifier could distinguish between the pattern of EEG response based on whether a participant *perceived* the stimulus as moving either towards or away in depth (based on their keyboard response during the task), regardless of the actual stimulus direction. This differed from our 'keyboard response' analysis where each mean epoch was formed by drawing from each of the four stimulus conditions to average out any stimulus information, rather than drawing from CD or IOVD data pools alone.

Here, we partitioned our data based on MID stimulus type and keyboard response. We bootstrapped four epochs that were *perceived* as heading towards in depth and four epochs that were *perceived* as heading away in depth. This was completed for CD and IOVD cues separately. These eight trials were then averaged together to create a mean epoch (i.e. a 'mean CD towards epoch' is formed by averaging four epochs from CD towards data that were *correctly identified* as moving towards in depth and four epochs from CD away data that were *incorrectly identified* as the participant perceived them as moving towards in depth). This was to ensure that each mean epoch was formed of an equal count of correct and incorrectly perceived responses. Identical to our main analysis, this was repeated 21 times to create 21 mean epochs for each of the four conditions (CD perceived towards, CD perceived away, IOVD perceived towards, and IOVD perceived away) for each of the 1000 bootstrapped iterations of the decoder.

We found that decoding occurred around 50% chance **(Figure S3)**. This suggests that successful decoding is based on the direction of the stimulus itself rather than its perceived direction or, critically, the preparation of the response-locked button press.

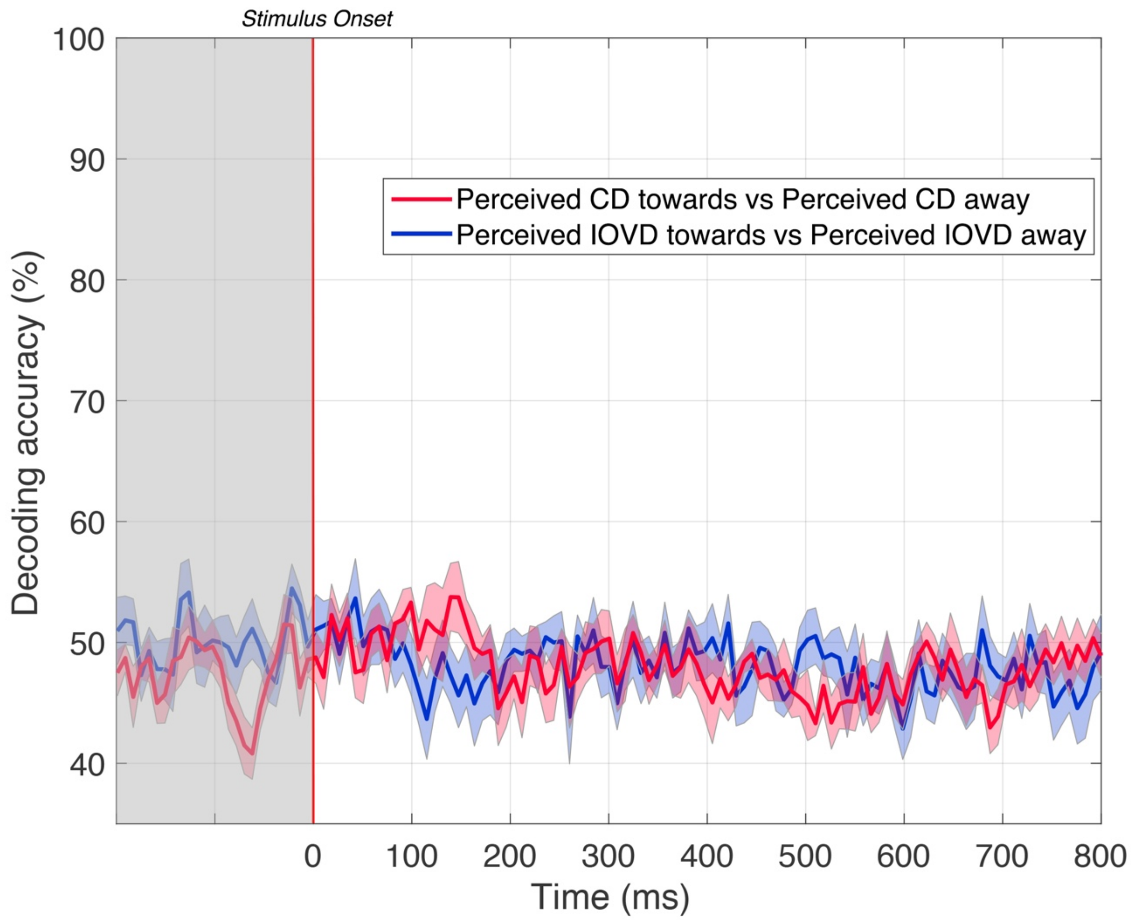

Figure S3. Pairwise decoding accuracies for comparing between data that was *perceived* as either moving towards or away in depth for CD and IOVD cues. Data is partitioned based on keyboard response, ($p < .05$). Shaded error bars represent ±1SE of the bootstrapped mean (1000 iterations).

Scalp distributions

We decoded EEG responses using all 64 electrodes across the scalp. Here, we present the grand average common average referenced EEG response amplitudes for each stimulus condition, averaged across all participants. In **Figure S4** we present mean scalp maps of the EEG responses both CD and IOVD cues, averaged across three time periods. The scalp maps show a strong positive voltage towards the occipital cortex during early stages of the EEG time course. During middle stages, this changes to strong negative voltage near the occipital cortex, and a spreading of response across the scalp. This continues in later stages of EEG response, where the distribution is generally similar, but responses are weaker.

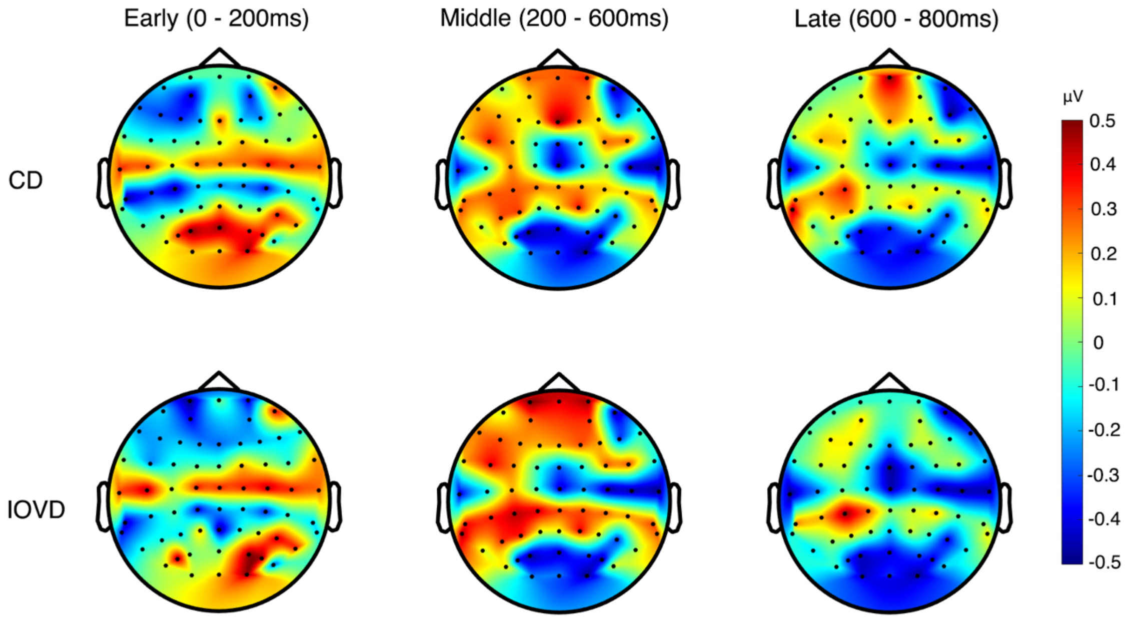

Figure S4. Scalp maps of mean common average referenced CD and IOVD (pooled across towards and away) EEG amplitudes are shown at three time periods – early (0 – 200ms post stimulus onset), middle (200-600ms post stimulus onset), and late (600 – 800ms post stimulus onset).

Similarly, in **Figure S5** we present scalp maps of the mean EEG responses both towards and away cues, averaged across three time periods. Again, these scalp maps show an initial strong positive voltage towards near the occipital cortex during early stages of the EEG time course, in middle stages this changes to a strong negative voltage near the occipital cortex, and the response distribution spreads across the scalp. This continues in later stages of EEG response, albeit with weaker responses.

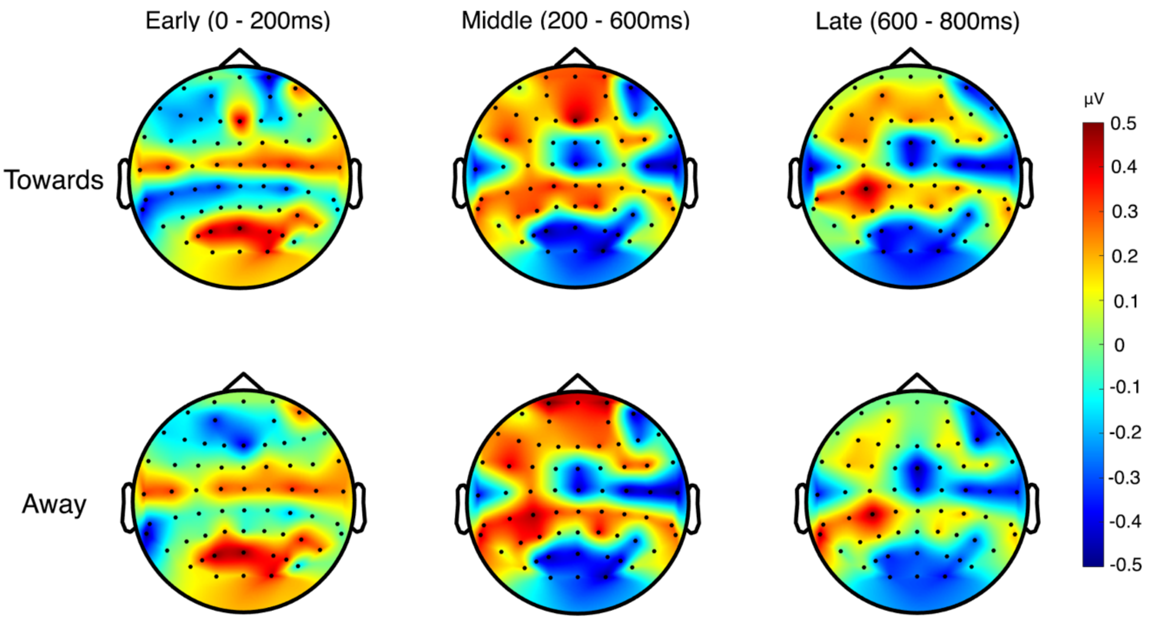

Figure S5. Scalp montages of mean common average referenced towards and away (pooled across CD and IOVD) EEG amplitudes are shown at three time periods – early (0 – 200ms post stimulus onset), middle (200-600ms post stimulus onset), and late (600 – 800ms post stimulus onset).

Decoding validation from shuffled labels

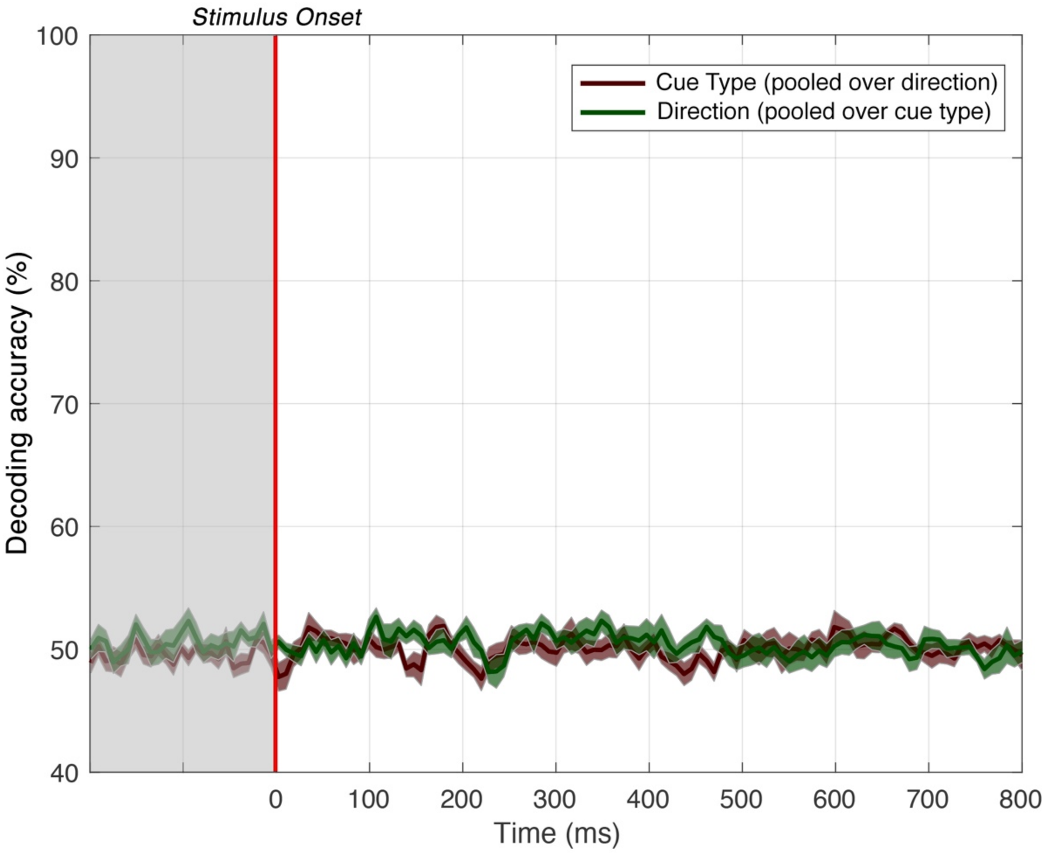

Figure S6. Shuffled label pairwise decoding accuracies for cue type (pooled over direction) in red and direction (pooled over cue type) in green. After shuffling labels, the decoding accuracy of both comparisons fell around the 50% (chance) baseline, confirming that the SVM was working as expected ($p < .05$). Shaded error bars represent ±1SE of the bootstrapped mean (1000 iterations).

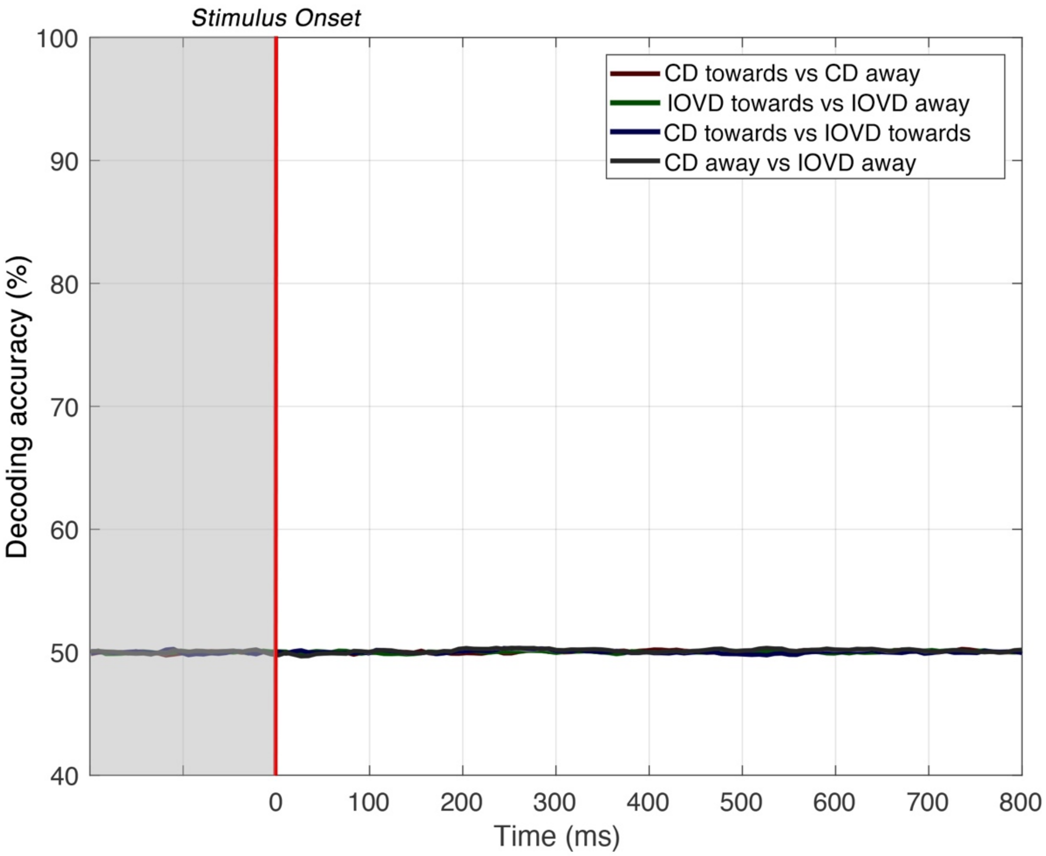

Figure S7. Shuffled label pairwise decoding accuracies for within cue and within direction conditions. After shuffling labels, accuracy for all four conditions was virtually identical, falling around the 50% (chance) baseline, confirming that the SVM was working as expected ($p < .05$). Shaded error bars represent ±1SE of the bootstrapped mean (1000 iterations).

Extended results of CD and IOVD discrimination within motion direction

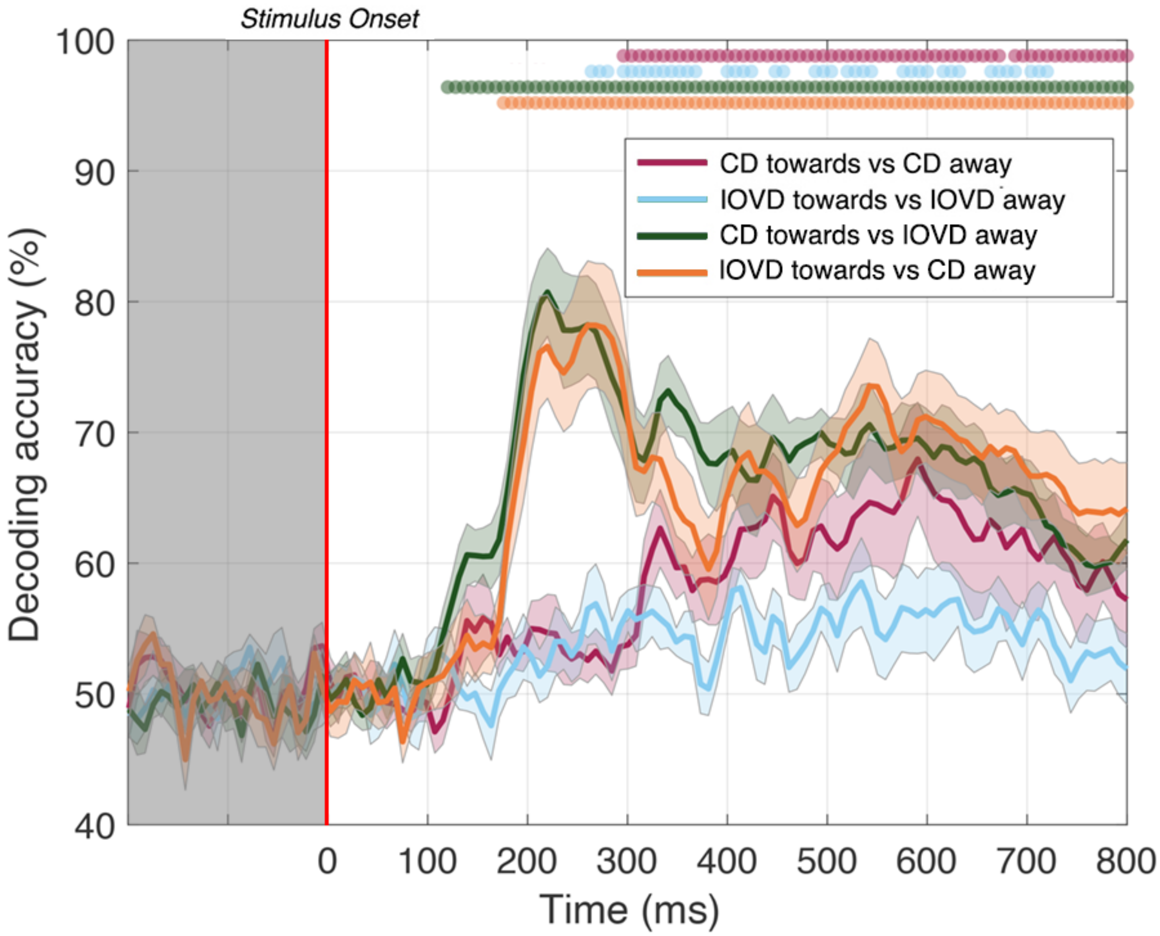

Figure S8. Pairwise decoding accuracy across 125 time points for four stimulus conditions. We included CD towards vs IOVD away and IOVD towards vs CD away to compare with decoding accuracy in **Figure 5**. These new comparisons do not particularly differ from those in Figure 5, suggesting that direction decoding cannot be disentangled from cue decoding, and that motion direction discrimination might be a shared process between cue types. Red, light blue, green, and orange dots indicate time points when the Bonferroni-corrected t-tests were significant for each condition ($p < .05$) and the coloured ticks on the x-axis represent the time point of peak decoding performance for each stimulus condition. Shaded error bars represent ±1SE of the bootstrapped mean (1000 iterations).
